# Longterm Protective Effects of Complement C3 Lowering in Adult APP‐KI Mice

**DOI:** 10.1002/alz.092944

**Published:** 2025-01-03

**Authors:** Brijendra Singh, Emma Spooner, Andre F. Batista, Takaomi C. Saido, Michael Carroll, Cynthia A. Lemere

**Affiliations:** ^1^ Brigham and Women’s Hospital; Harvard Medical School, Boston, MA USA; ^2^ Brigham and Women’s Hospital, Boston, MA USA; ^3^ RIKEN Center for Brain Science, Wako, Saitama Japan; ^4^ Boston Children’s Hospital; Harvard Medical School, Boston, MA USA

## Abstract

**Background:**

Previously, we found that germline C3 deletion protected cognition and hippocampal synapses in aged APP/PS1dE9 mice, despite increasing Aß plaques. Here, we crossed our C3 inducible conditional mouse model to APP^NL‐G‐F/NL‐G‐F^ knockin mice to determine whether global C3 lowering in an adult amyloid mouse model would be protective.

**Methods:**

C3^fl/fl^;Rosa26‐Cre‐ERT2 (C3iKO) mice were crossed to C3^fl/fl^;APP^NL‐G‐F/NL‐G‐F^ mice to generate APP;C3iKO mice, which received 75 mg/kg tamoxifen (TAM; n = 16) or corn oil (CO; n = 15) for 5 days at 3.6 months of age. Serum was collected after 30 days and at the termination of the study to measure C3 protein levels by ELISA. Behavioral testing was conducted at 15 months after which the mice were euthanized, and brain tissue harvested. ELISAs, immunofluorescent staining, qPCR and unbiased RNAseq of prefrontal cortical tissue were performed.

**Results:**

Serum C3 levels were significantly reduced ∼85% 30 days post‐TAM and ∼70% at the end of the study. The TAM‐treated APP;C3iKO mice performed significantly better on the Spatial Novelty Y Maze, Novel Object Recognition and Novel Object Location tests compared to CO‐treated mice. C3 mRNA expression and protein levels in brain were significantly reduced; C1q protein levels were also reduced in brain. No difference in overall amyloid load was seen by IHC or ELISA in APP‐KI;C3iKO mice. We found no changes in overall Iba‐1 and GFAP immunoreactivity between the two groups. However, plaques were associated with fewer Iba‐1+/CD68+ microglia and AQP4+/GFAP+ astrocytes in the TAM‐treated animals. PSD‐95 post‐synaptic staining was increased in hippocampal CA3 but no difference was seen in SYP pre‐synaptic staining. C3 lowering in the APP‐KI mice resulted in significantly reduced mRNA expression of C3, TNFa, IL‐10, CX3CR1 and AQP4 but only a trend for reduced C1q mRNA by qPCR. Unbiased bulk RNAseq of prefrontal cortex identified 1071 differently expressed genes (DEGs) including 569 upregulated genes and 502 downregulated genes in the APP;C3iKO mice. Further analysis of these DEGs is underway but thus far, many of the top hits are related to synaptic signaling.

**Conclusion:**

Our data indicates that global C3 lowering in adult APP‐KI mice reduced inflammation and protected synapses and cognition.

**Funding**: NIH/NIA RF1 AG060057 (CAL)

